# Analysis of Transition of Patients with Parkinson’s Disease into Institutional Care: A Retrospective Pilot Study

**DOI:** 10.3390/brainsci11111470

**Published:** 2021-11-06

**Authors:** Ida Jensen, Emily Lescher, Stephanie Stiel, Florian Wegner, Günter Höglinger, Martin Klietz

**Affiliations:** 1 Department of Neurology, Hannover Medical School, 30625 Hannover, Germany; emily.borns@stud.mh-hannover.deb (E.L.); Wegner.Florian@mh-hannover.de (F.W.); Hoeglinger.Guenter@mh-hannover.de (G.H.); Klietz.Martin@mh-hannover.de (M.K.); 2 Hannover Medical School, Institute for General Practice and Palliative Care, 30625 Hannover, Germany; Stiel.Stephanie@mh-hannover.de

**Keywords:** Parkinson’s disease, caregiver burden, transition

## Abstract

Parkinson’s disease (PD) is a neurodegenerative disease which gives a person a high risk of becoming care-dependent. During disease progression, the amount of care concerning activities of daily living can increase, possibly resulting in transition of the people with Parkinson’s disease (PwP) to a care facility. However, there is a lack of knowledge concerning the factors leading to institutionalization of PwP and the consequences for them and their informal caregivers. The aim of this cross-sectional retrospective study was to investigate reasons leading to the transition into an institutional care facility, the process of decision-making and its effects on PwP symptoms and caregiver burden. Participating PwP had to be institutionalized for at most one year after transition at study inclusion. Participants completed a range of semiquantitative questionnaires as well as the caregiving tasks questionnaire. Fourteen patient–caregiver pairs were included. PwP suffered from late-stage PD symptoms with high dependence on help, experiencing several hospitalizations before transition. Analyses revealed a significant decrease in caregiver burden and depressive symptoms of the caregivers after PwP institutionalization. Factors influencing the transition were, e.g., fear of PwP health issues and concerns about caregivers’ health. This study presents new insights into the process of institutionalization and its influence on caregiver burden, including aspects for discussions of physicians with PwP and their caregivers for counselling the decision to move to institutional care.

## 1. Introduction

Parkinson’s disease (PD) is a progressive movement disorder constraining the lives of people with PD (PwP) and their relatives [1]. Over the course of the disease, the cardinal motor symptoms of bradykinesia, tremor at rest, rigidity and postural instability progressively worsen [2]. Additionally, non-motor symptoms such as depression, cognitive decline, anxiety, constipation and urinary-urge inconsistency can occur, placing an additional burden on PwP and their relatives [3,4,5,6]. Those relatives, mostly spouses, often become informal caregivers over the course of the disease. As shown in recent research, caring for PwP can cause mental and physical distress, resulting in a reduced quality of life and caregiver burden [7,8,9,10]. It contains straining negative mental, physical and socioeconomic consequences resulting from caring for a person living with a chronic progressive disability [11]. Caregiver burden in PD is a global issue, regardless of geographical, geopolitical and cultural differences [7,8,9,10]. Given the fact that a larger number of people attain a great age, more cases of PD and consecutive late-stage PD will appear over the next decades [12]. Therefore, the demand on informal caregivers will increase. It is also known that a higher dependence of patients can correlate with an increased burden on the informal caregiver [13]. In advanced stages of PD, care at home becomes too debilitating, and caregiver burden increases dramatically. Consequently, the question of transition of the PwP into an institutional care facility may arise. Up until now, there has been a large gap of knowledge about the main factors leading to institutionalization. Moreover, the process of decision-making concerning PwP is still unclear and needs further evaluation. Given the fact that due to an aging population, the cases of PD will rise, the influence of transition on the PwP and the caregiver burden will become an increasingly important issue in the next decades. Therefore, the examination of effects of transition on both PwP and their informal caregivers is a needed investigation.

The aim of the present pilot study was to elucidate the process of decision-making concerning the transition of PwP into institutional care and retrospectively describe the effect of institutionalization on PwP and their informal caregivers, especially the caregiver burden.

## 2. Materials and Methods

### 2.1. Participants

The research protocol was approved by the local Ethics Committee of Hannover Medical School (Ethics-ID: 3123-2016; Amendment in 2020). All participants gave written informed consent in accordance with the Declaration of Helsinki. Our sample included 14 PD patients and their 14 caregivers. Recruitment took place from August until December 2020. Data was collected exclusively for this study. The patients included in this study met the Movement Disorder Society (MDS) clinical diagnostic criteria for PD [14]. PwP had to be institutionalized in a care facility for at most one year at study inclusion (t1). Baseline data (t0) concerning the care situation at home before transition were assessed retrospectively. PwP and their caregiver were asked to describe the situation in the last week at home before the transition into institutional care as a baseline assessment (t0). The evaluation after transition was performed at the time of study participation (t1). The maximum possible interval between t0 and t1 was one year. This limit was set to avoid memory bias of the participants. Criteria for selection of caregiver participants included being the primary caregiver and managing most of the caregiving time with the patient. Professional caregivers were not included because of other coping mechanisms and their professional training in these situations. Dropouts of the study were either not contactable, the caregiver also had been transferred into institutional care or the caregiver was not able to complete the questionnaire. In total, six PwP and their caregivers dropped out of the study. Patients and caregivers did not receive financial compensation for participating in the study. Data on the PwP were mostly provided by the caregiver, but if possible, from PwP and caregiver together. Completion of the questionnaires took about 40 min for each patient and the informal caregiver.

### 2.2. Measures

An overview of the accessed questionnaires is presented in Table A1. To measure the patients’ PD impairments, the Hoehn and Yahr stage, ranging from a minimum of 1 point (unilateral symptoms) to a maximum of 5 points (confinement to bed or wheelchair) [15], was estimated before the transition into the institutional care and afterwards. Further, the disease duration and, to measure dependence range, the German federal insurance care level (ranging 1–5) before transition were noted. To gather information on external dependence of the PwP, the Barthel Index, ranging from 0 to 100 (completely independent) points before and after the transition, was assessed [16].

In addition, the number of times the patients were admitted to a hospital before and after transition was assessed as well as falls before and after the transition, and the consequences of the severest fall with regard to resulting injuries were investigated. Further questions addressed the outpatient connection to a neurologist and if a lasting power of attorney, living will or advance directive existed.

The care situation before the transition was assessed as follows: Caregivers were asked to state if they were the only relatives caring for the patients; if a care service provided additional support, and if so, which tasks were taken over; and if physical, speech or occupational therapy was attended either at home or in a practice. Sociodemographic information about patients and caregivers including age, relationship status, education, job, daily amount of time spent together and time dedicated to the patients’ care before and after transition were collected as well as the physical condition of the caregivers before PD onset of the patient and while caring for the patient before and after transition.

### 2.3. The Process of Transition into Institutional Care

To gather information about the transition, patients and caregivers were requested to divulge who primarily made the decision. Who was involved into the decision-making process? How long did the decision-making process take? How long did it take from the rapid worsening of care at home till transition into nursing home? Had a particular nursing home been taken into consideration before the transition?

### 2.4. Influence of Transition into an Institutional Care on Caregiver Burden and Tasks

Different caregiver burden questionnaires were administered. One part of the validated German version of the Parkinson’s disease caregiver burden questionnaire (PDCB), the global burden scale, was handed out to the caregivers. The definition of global burden was explained to the participants in comprehensive form in German. They were asked to rate their global burden as a caregiver on a scale from 0 (no burden) to 100 (maximum burden) [17]. Additionally, participants reported on visual analogue scales how much they were strained by organizing therapies (e.g., physiotherapy, occupational therapy, logotherapy) (from 1 = no burden to 10 = heavy burden) and how much the patients were burdened by the therapies (from 1 = no burden to 10 = heavy burden). 

Furthermore, caregivers ranked their general fear of the future on a scale from 0 (no fear) to 100 (strong fear of the future) before the transition and currently. Additionally, caregivers could choose reasons for fear of the future before and after transition, such as guilt or fear of health issues.

The adapted caregiver task questionnaire was assessed to seek out specific caregiving tasks and how they were affected by the transition [10]. It contains 12 items describing different caregiver tasks and one item free to adapt for own modification. Each item has a score ranging from 0 (no burden) to 10 (unbearable burden). The total score of the caregiver burden results from the sum of the individual items ranges from 0 to 120 points. Additionally, participants could define how much time they spent on each task per week. Concerning mood state, both patients and caregivers were asked to rate symptoms of depression and sadness on a scale from 0% (no sadness/depression) to 100% (heaviest depression) before and after the transition into the institutional care. 

### 2.5. Analyses

Data were presented in mean and standard deviation (SD) for descriptive analyses. Analyses were performed using IBM SPSS 25.0. A t-test was used to determine the significance of changes between t0 (time short before transition, max. one year before transition to institutional care) and t1 (state after transition, maximum 1 year in institutional care). A *p*-value below 0.05 was considered statistically significant. Because of the exploratory nature of the study and the low number of participants, no multiple comparison correction was performed.

## 3. Results

### 3.1. People with Parkinson’s Disease and Caregiver Characteristics

Demographic and clinical characteristics of PwP (n = 14, 43% females) and caregivers (*n* = 14, 71.4% females) are presented in Table 1.

On average, PwP were 79 years old (±5.8; range 72–88) with a disease duration of 15.4 years (±9; range 5–34 years). The average Hoehn and Yahr stage of 4.1 (±0.5; range 3–5) worsened slightly after transition to institutional care with a mean stage of 4.5 (±0.6; range 3–5), indicating advanced stage of PD.

A total of 78.6% (*n* = 11) of the caregivers were spouses living with the PwP. Concerning the remaining caregivers, two were daughters and one was a sister of the PwP. The mean caregiver age was 73.2 years (±9; range 48–86). The majority of caregivers were the only caregiving relatives; only 3 out of 14 caregivers had additional support by other relatives.

### 3.2. Properties of the Homebound Phase and Changes after Transition to Institutional Care Facility

All PwP were treated by a neurologist, and 64.3% went to see their neurologist at least one time within four months. Before transition to institutional care, 71.4% of the patients were also supported by a professional care service at home. The main tasks of these professional care services were helping with personal hygiene, e.g., showering, clothing as well as mobilizing and sometimes providing medicine. All patients received regular physiotherapy, 78.6% additional speech therapy and 64.3% occupational therapy. The burden caused by getting to and receiving therapies was perceived almost equally with a score of 4.4 ± 2.5 for patients and 4.9 ± 3.2 for caregivers (*p* = 0.3). Thirteen patients had a living will and lasting power of attorney, and 11 had advance directives.

Overall, the time before institutionalization was characterized by several hospitalizations and falls. About 57.1% of the patients were hospitalized at least two times in the course of the year before transition into the facility care. About two thirds (69%) of the patients fell more than three times at home, subsequently suffering from injuries with consecutive doctor contact or hospitalization. The number of assessed patients with more than three severe falls decreased after institutionalization (33%), as well as the severe consequences (41.6%).

The dependence on external help in daily activities and immobility was rather high, indicated by a low Barthel Index of 37.5 (±20.1; range 10–100) before and 27.5 (±9.7; range 10–40) after transition, indicating a significant drop within one year (*p* = 0.02). Clinical worsening was also suggested by the increase in the German federal care insurance level from third to fourth grade. The patients reported moderate depressive symptoms (45 ± 20.3; range 0–70) which did not change significantly after transition to institutional care facility (40.9 ± 27.5; range 10–100; *p* = 0.4).

### 3.3. Factors Contributing to the Decision Process Leading to Institutionalization

Concerning the process of transition, all caregivers were involved in decision-making or initiated it. In 43% of the cases the PwP were directly involved in the decision process; hence, in the other cases, the PwP were too severely affected by the disease to actively discuss this issue. Further, in 64% of the cases, physicians contributed to the decision-making. In 55% of the cases, this process was performed in the afterword of a hospital stay, because the care could not be delivered in a homebound setting anymore. Others involved in the decision-making for an institutional care facility included neurologists in 44%, general practitioners in 22% and professional caregivers such as nurses in 11% of the cases. On average, the decision-making took up to 6–12 months, and 3–4 months were needed for the transitioning. As reasons contributing to the decision (multiple answers possible), 10 caregivers reported the patient’s health, 10 mentioned being overwhelmed by caregiving and 9 added their own health as a factor. After transition, five caregivers still reported fear of the health of the patient, four were concerned about their own health and no participant feared of being overwhelmed. In total, six caregivers reported feelings of guilt because of the transition of the PwP to an institutional care facility. Fear of financial needs was mentioned two times before and three times after transition by the caregivers.

### 3.4. Effects of the Institutionalization on Caregiver Measures

Caregivers reported significantly less burden after the patients’ transition to an institutional care facility. As displayed in Table 2, the average time spent together decreased from 128.5 h (± 7.1; range 7–168) per week, of which 53.3 h (± 49.8; range 0–168) per week were dedicated only for care, to 17.3 h (±20.7; range 0–70) spent with the patient after the transition, of which 10.1 h (±15.8; range 0–56) were spent in the caregiving role, indicating a significant drop in general time spent together (*p* < 0.001) and in time for caregiving (*p* = 0.007).

Results of the caregiver task-questionnaire are displayed in Table 3. The time spent for specific caregiver tasks decreased significantly from 67.8 ± 39.8 to 24.4 ± 23.7 h per week, indicating a drop of 36% (*p* = 0.002). The assessed burden of caregiver tasks in total decreased significantly from 57.6 ± 19.6 to 20 ± 7.1 (*p* < 0.001). Before transition, caregivers felt most burdened by transportation of the patient, followed by help with personal hygiene, help during the night and providing food and drinks. The burden dropped significantly in all assessed subcategories except emotional support (*p* = 0.21) and other tasks described by the caregivers, indicating a major relief of burden concerning general care after the transition.

The measured decrease in perceived burden was also reflected in the PDCB global burden, which indicated severe caregiver burden (76.4 ± 21.2%; range 30–100%) at baseline. It improved significantly after transition of the PwP to an institutional care facility, displaying only mild to moderate caregiver burden (28.3 ± 26.1%; range 0–100%; *p* < 0.001).

The depressive symptoms of caregivers decreased significantly with a score of 47.1 (±28.9; range 0–100) before the transition to 34.2 (±25.6; range 0–100; *p* = 0.04) afterwards. Concerning fear of the future, no significant difference could be measured (*p* = 0.1).

Before the onset of PD in their patients, no caregiver reported health issues. During disease progression, 43% of the participants reported psychological or physical difficulties. After transition, five caregivers out of fourteen reported psychological or physical issues. When asked about fear of the future, 42.9% of caregivers rated it as relevant on average (±22.2; range: 0–80%) before and 29.2% after transition (±26.9%; range 0–100), indicating no significant change (*p* = 0.095).

## 4. Discussion

In this cross-sectional pilot study, the transition of PwP into an institutional care facility was analyzed. Included patients suffered from late-stage PD with a high dependence on external help. Additionally, depressive symptoms were reported, which are commonly associated with late-stage PD [18]. Severe falls, which are one major complications of advanced PD [19], occurred frequently. The decreased mobility of PwP can lead to irregular meetings with neurologists, fewer physical therapies [3,20] and might be one reason for the often insufficient treatment in late-stage PD [19]. As patients suffered from advanced PD symptoms and dependence on care, caregivers reported a high burden associated with caregiver tasks and depressive symptoms. When living at home, the caregiver automatically becomes the main support for PwP. Subsequently, the burden perceived by the caregiver is higher when PwP still live at home, and an increasing burden is an important predictor for institutionalization [21,22]. In total, 71% of the PwP required support by professional care services before the transition to institutional care. The increasing dependence on help can force the PwP to plan their days based on the official care appointments, sometimes waiting hours to be taken care of [23]. With progressing disease, PwP are more likely to live in a nursing home which can provide care at all times [24].

The ongoing disease requires housing adaptions and equipment. Those can become expensive and are sometimes impossible for the families to afford [25]. In general, the estimated costs of informal care and burden of caregivers are the highest in the late stages of PD [24]. Eventually, both caregiver and patient realize that living at home is not safe and sufficient anymore and that a care facility could be an improvement for daily living, feeling safe and no longer having to worry about future care supplies [26,27,28]. 

### 4.1. The Progress of Transition into Residential Care Facilities

The results of this study display a prolonged progress of decision-making concerning transition to institutional care of at least 6 to 12 months, which reflects the complexity of the process. The transition into a nursing home is a major life event which requires elderly people to adapt to a new environment, leaving their former life and independence behind [29,30,31]. Groenvynck et al. investigated major factors which influence the transition into nursing homes and divided the transition process into three phases: the pre-transition phase, the phase of moving into the institutional care facility and the final part of the transition, the admission [32].

Current international literature reflects that in the pre-transition phase, PwP and caregivers wish to form a triad including a healthcare professional for guidance and decision-making [33]. The same tendency is displayed in our data: even though caregivers were always and PwP were partly involved in the decision progress, in only four cases, the caregiver and patient made the decision alone. Neurologists, general practitioners and care staff were mostly involved in the decision-making. Those results reflect the need for professional knowledge and estimation of the care situation by external specialists. As described for dementia, which often occurs in late-stage PD, patients often are too busy mastering their actual daily life and do not spend time and energy on planning the future. Additionally, with progressing dementia, PwP often become too ill to take part in the decision-making process [34].

Additionally, typical psychiatric symptoms, such as depression, apathy and avoidance of novelty [35] or alexithymia, might come across as disinterest and hinder the decision progress [36]. Therefore, as far as late stages of neurodegenerative diseases are concerned, caregivers have to take on the responsibility of decision-making on their own [37]. Similar findings were reflected in this study, as all caregivers were involved in the decision of transition or initiated it. Only in 43% of the cases were the PwP directly involved in the decision process; hence, in the other cases, the PwP were too severely affected by the disease to actively discuss this issue.

Consequently, caregiver burden can increase due to the decision process. Even though caregivers seem to recognize the need of transition earlier than the PwP, they generally do not want to urge them [38]. Eventually, the feeling of togetherness and love can reduce the burden of PD and its progress for a certain time [25]. Typically, the decision for transition of the beloved relative is the “last resort”, causing feelings of sadness, loss of control and guilt in the caregiver [25,38,39]. Therefore, the feeling of being prepared for this change seems to be a major aspect improving the satisfaction with transition [39,40]. It is recommended to have annual checkups with patients and their caretakers to establish plans concerning the future [41].

Concerning the second phase which refers to the actual process of moving, PwP and caregivers need enough time to mentally prepare for the upcoming institutionalization [32]. As our data displayed, the process of moving could take up to an additional 3–4 months. Getting to know several nursing homes in order to choose a suitable residency is considered important [42,43,44]. A rushed or unexpected transition can leave patients and caregivers overwhelmed and stressed, as it is the case when the transition happens after hospitalization [27,45]. 

Unfortunately, severe falls of PwP increase with disease progression [46]. In this study, 69% of the patients suffered from severe injuries after falls with consecutive doctor contact or hospitalization. In 55% of the cases, the decision for transition was implemented after a hospital stay, as care could not be secured/delivered in a homebound setting anymore. 

The final part of transition concerns the admission and establishment of a new feeling of home [32]. Moving can restrict self-efficacy which is associated with a decreased life satisfaction in late-stage PD [47]. Therefore, patients should be supported in keeping their autonomy to increase well-being in a residential care facility setting [48] and of independence activities in daily living [49]. Factors leading to an improved transition can be keeping personal possessions, continuing relationships and finding new ones in the facility [42].

### 4.2. The Influence of Transition to Institutional Care on Patients with Parkinson’s Disease and Their Caregivers

During the course of one year, PD symptoms of the study participants increased: patients became more reliant on external help. Surprisingly, our data did not display a significant influence of transition on the patients’ depressive symptoms. As previously reported, depressive symptoms stayed on an equal level. This could, on the one hand, be explained by a progressing indifference of the environment and apathy caused by PD [50]. On the other hand, Kahn et al. pointed out that elderly people in general tend to downplay negative aspects of nursing homes, rationalize their transition and realize that by living in a nursing home, care can be provided best [51].

The number of assessed patients with more than three severe falls decreased after institutionalization (33%), as well as the severe consequences (41.6%). In an assessment of 90 PwP (average age 81.3 years), the number of emergency department attendances, hospital admissions and length of hospitalization were significantly lower when patients lived in a care facility than when previously living at home [52]. Considering those results, nursing homes can be a safer environment for people with late-stage PD than the former home [53]. However, Walker et al. recently still determined falls as the prominent cause for attending emergency departments. Therefore, staff should be trained to handle gait problems and symptoms of late-stage PD more professionally to avoid incidents [54]. 

The most striking effect of transition was observed concerning caregiver burden. The transition seemed to disconnect the association of increasing caregiving burden during disease progression, indicating a strong relief of burden. Time spent for caregiving tasks decreased significantly. Consequently, depressive symptoms decreased significantly as well. Only feelings of guilt and fear of the future did not change significantly, although guilt may be aggravated through transition, which is described in the literature [25]. 

Since time spent for caregiving tasks decreased significantly, caregivers had more energy to spend on emotional support and quality time with the PwP [25]. Caregivers express a wish to stay actively involved in the life of their relatives [55]. As caregivers gained a unique understanding of the individual symptoms and treatment of PwP over the course of the years, they have to be included in therapeutical decisions after transition as well [56]. The process of role changing during transition concerning caregivers should be analyzed further in the future. 

### 4.3. Future Aspects for Transition of Patients with PD in Residential Care Facilities

A recent German study investigated the average amount of PwP in nursing homes: the prevalence rate of residents with PD was 13.9%. Even though those PwP received regular medical consultation and treatment, neither care by specially trained nursing staff nor a central coordination of care was reported [57]. An adjustment in the future could be to establish networks with physicians, especially trained staff, therapists, caregivers educated in the treatment of PD and PD nurses to improve healthcare and reduce healthcare expenditures [57,58]. If the residency provides specialized staff, therapists and doctors, this might improve the PwP’s and caregivers’ situation significantly, since special knowledge on PD is reported to increase quality of life, symptoms and disease progression [52]. Knowledge of the disease and cooperation of neurologists in hospitals with residential care facilities can improve the course of the disease and should be further improved [59]. Despite the helpful role of PD nurses, those are not available in several countries, which needs to be improved in the future [60].

As reflected in our data, the transition into a nursing home can be a massive relief for the caregivers. Because the cases of late-stage PD are going to increase during the next years and a trend towards a more personalized care evolves [61], research about more specialized nursing homes and their influence on disease progression and satisfaction, as well as its influence on the caregivers, is needed.

## 5. Conclusions

So far, there is limited scientific knowledge addressing the transition into an institutional care facility of PwP. As this study implicates, transition into institutional care takes part mainly in advanced stages of PD. Advanced symptoms were associated with high caregiver burden and moderate depression of informal caregivers. The decision of moving to an institutional care facility was predominantly suggested and carried out by the caregiver and took several months, also because a large proportion of the PwP was not able to participate in the process of decision-making due to their advanced disease state. After institutionalization, caregiver burden and depressive symptoms were significantly reduced. 

Consequently, the possibility of transition into institutional care should be addressed and discussed much earlier than it actually seems to be the case. Caregivers and PwP should be guided and supported by PD specialists to meet their specific disease-related needs. Therefore, clinicians’ awareness of the issue of caregiver burden should be increased. Informal caregivers should be informed about supporting options concerning care much sooner to improve the care supply at home. This way, caregiver burden concerning the time before transition of PwP could be reduced. With ongoing disease progression, the transition as a possibility to relieve informal caregivers and improve the care conditions of the PwP should be supported. Consequently, the supply of specialized care and networks to support PwP and their caregivers over the course of the disease has to be discussed and improved. Hence, further studies including more participants are needed to investigate the influence of transition on the PwP and the caregiver burden which will become an increasingly important issue in the future. 

## 6. Limitations

The main limitation of this study surely is the limited number of participating PwP and their caregivers. However, recruitment of these specific PwP is extremely difficult because of the advanced PD symptoms, cognitive decline and care dependency. Moreover, most caregivers of these patients were not available for participating in such a study. Contrary to these limitations, these data are important for general physicians and neurologists treating people with advanced PD, both at home and in care facilities. 

To avoid bias in the retrospective part of data collection, we used several visual analogue scales and did not use a specific instrument for the measurement of depressive symptoms. This more general measurement was done to avoid false results and has to be replaced by a specific scale in longitudinal studies. Additionally, as the questionnaires were accessed retrospectively and were completed by the participants at home, we could not directly access the cognitive function over the course of the transition. Nevertheless, the influence of transition on cognitive function of PwP could be considered an interesting aspect of investigation in the future. A selection bias cannot be excluded, because people more interested in this topic were more likely to participate in this study. 

## Figures and Tables

**Table 1:** Patient (*n* = 14, female = 6) and caregiver (*n* = 14, female = 10) characteristics before and after transition to institutional care.

	**Mean ± SD**	**Min**	**Max**
**PD Patients**			
Age (years)	79.1 ± 5.8	72	88
Disease duration (years)	15.4 ± 9	5	34
Hoehn and Yahr stage before	4.1 ± 0.5	3	5
Hoehn and Yahr stage after	4.5 ± 0.6	3	5
Burden because of therapies	4.4 ± 2.5	1	8
Depression before	45 ± 20.3	0	70
Depression after	40.9 ± 27.5	10	100
Barthel Index before	37.5 ± 20.1	10	100
Barthel Index after	27.5 ± 9.7	10	40
	**Mean ± SD**	**Min**	**Max**
**Caregivers**			
Age (years)	73.2 ± 9	48	86
Caregiving hours before (h/w)	53.3 ± 49.8	0	168
Caregiving hours after (h/w)	10.1 ± 15.8	0	56
Time together before (h/w)	128.5 ± 7.1	7	168
Time together after (h/w)	17.3 ± 20.7	0	70
Burden because of therapies	4.9 ± 3.2	0	10
PDCB before (%)	76.4 ± 21.2	30	100
PDCB after (%)	28.3 ± 26.1	0	100
Depression before	47.1 ± 28.9	0	100
Depression after	34.2 ± 25.6	0	100
Fear of the future before (%)	42.9 ± 22.2	0	80
Fear of the future after (%)	29.1 ± 26.9	0	100

PwP disease duration was measured at time of participation in the study t1. Abbreviations: h/w, hours per week; PD, Parkinson’s disease; PDCB, Parkinson’s disease caregiver burden questionnaire; SD, standard deviation.

**Table 2:** Comparison of patient and caregiver characteristics before and after transition.

	**PwP**	**Caregiver**	***p***
Burden because of therapies	4.4 ± 2.5	4.9 ± 3.2	0.3
	**Before**	**After**	***p***
Time spent together (h/w)	128.5 ± 7.1	17.3 ± 20.7	<0.001 *
Time spent for care (h/w)	53.3 ± 49.8	10.1 ± 15.8	0.007 *
Burden of caregiver in total	57.6 ± 19.6	20 ± 7.1	<0.001 *
Time spent for caregiving tasks (h/w)	67.8 ± 39.8	24.4 ± 23.7	0.002 *
Depression patient	45 ± 20.3	40.9 ± 27.5	0.4
Fear of the future (%)	42.9 ± 22.2	29.1 ± 26.9	0.1
Barthel Index	37.5 ± 20.1	27.5 ± 9.7	0.02 *

Abbreviations: h/w, hours per week; * *p* ≤ 0.05.

**Table 3:** Change of caregiving task questionnaire results before and after transition.

	Before (*n* = 13) Mean ± SD	After (*n* = 12) Mean ± SD	*p*
Shopping	3.1 ± 2.2	0.8 ± 1.0	0.004 *
Housekeeping (e.g., cleaning, ironing)	4.1 ± 1.8	1.6 ± 2.2	0.007 *
Preparing/serving of meals	5.2 ± 2.5	1 ± 1.2	<0.001 *
General body care (e.g., dressing, toilet, showering)	5.8 ± 2.8	0.8 ± 1.1	<0.001 *
Mobilizing/positioning the patient	4.3 ± 3.1	0.5 ± 1.0	0.004 *
Helping during the night	5.5 ± 4.0	0 ± 0	<0.001 *
Alleviating symptoms (e.g., on-demand medication)	3.5 ± 2.3	0.2 ± 0.6	<0.001 *
Providing social/emotional support	4.2 ± 2.8	3.7 ± 2.3	0.21
Collecting information	4 ± 3.0	2.1 ± 2.2	<0.001 *
Taking part in decision-making	4.9 ± 2.7	2.6 ± 2.1	0.009 *
Administrativ741e tasks (e.g., health insurance)	4.2 ± 3.1	2.1 ± 1.2	0.006 *
Taking/accompanying the patient, e.g., to the doctor/therapies	6 ± 2.7	1.5 ± 2.2	0.001 *
Other	0.7 ± 1.7	1.6 ± 1.8	0.21

Hence, one caregiver did not complete the caregiver tasks questionnaire at t0 and two caregivers at t1. Abbreviations: SD, standard deviation; *, *p* ≤ 0.05.

## Data Availability

Data were available on reasonable request to the corresponding author.

## Appendix A

**Table A1:** Overview of the completed questionnaires before and after the transition.

Questionnaire	Timepoints of Assessment	
	Before	After
**Answered by caregivers concerning themselves**		
General demographics of caregiver		X
Time spent with PwP	X	X
Time spent for caregiving	X	X
Caretaker situation (e.g., other caregivers, professional support)	X	X
Caregiver task questionnaire	X	X
PDCB global burden scale	X	X
Depression scale	X	X
Burden because of PwP’s therapies	X	X
Fear of the future	X	X
**Answered by PwP and caregivers concerning PwP**		
General demographics of PwP		X
Hoehn and Yahr stage	X	X
Times of hospitalization	X	X
Times of falls	X	X
Consequences of the severest falls	X	X
Barthel Index	X	X
**Care supply**		
German federal insurance care level	X	X
Applied physical therapies (physiotherapy, occupational therapy, logotherapy)	X	X
Burden of therapies	X	X
Did lasting power of attorney, living will, advance directive exist?		X
**Transition process**		
Questionnaire on the decision for transition process		X
